# The Treatment of Pleurisy

**Published:** 1892-11-19

**Authors:** 


### The Hospital Clinic.

[*The Editor will be glad to receive offers of co-operation and contributions from members of the profession. All letters should be addressed to* The Editor, The Lodge, Porchester Square, London, W.]

### BROMPTON CONSUMPTION HOSPITAL.

### The Treatment of Pleurisy.

The treatment of acute pleurisy as employed at the Brompton Consumption Hospital may be conveniently divided into three stages, in accordance with the pathology of the disease, viz.: (1) The treatment of the stage of hyperæmia, (2) the treatment of the stage of effusion, (3) the treatment of the stage of absorption.

*Treatment of the Stage of Hyperœmia.*—The chief objects before us in the treatment of this stage of the disease are: (*a*) The relief of pain, (*b*) the diminution of the inflammation and the pyrexia. The patient must, even in mild cases, be confined to bed, and the diet must consist entirely of fluids. Thirst may be assuaged by bland fluids, which need not be restricted in amount. Stimulants are to be avoided unless specially required. For the relief of pain the application of from four to eight leeches, according to the condition, age, and sex of the patient, is generally sufficient. Hot poultices or turpentine stupes are used for the same purpose. Dr. Powell recommends a blister under the poultice as useful in some cases. The subcutaneous injection of one-quarter or one-third of a grain of morphine may be tried, if the above measures fail to relieve the pain. In mild cases of acute pleurisy, and in that form known as "dry pleurisy," Dr. Roberts' plan of strapping the affected side firmly is the most efficacious for the relief of pain, and the one usually adopted at Brompton. The bowels should be kept freely open by the use of salines and an occasional mercurial purge, *i.e.*, calomel (gr.i.-iii.) It is best to commence the medicinal treatment by giving a dose of calomel (gr. ii.), followed by a saline. The action of the skin and kidneys should be promoted by the use of such drugs as the acetate and nitrate of potassium and full doses of liquor ammonii acetatis (ʒii.-ʒiv.). Dr. Powell recommends the use of small doses of aconite *before* the commencement of effusion. The temperature in pleurisy rarely rises sufficiently high to call for special treatment. Sleep may be obtained by the use of some preparation of opium, best given in the form of Dover's Powder.

*Treatment of the Stages of Effusion and Absorption.*—When effusion has occurred, and provided that the pyrexia continue, the treatment already employed during the first stage should be continued unaltered for at least fourteen days. During this period no attempt to remove the fluid by operation should be made. If, however, the temperature falls, absorption should be aided by the use of counter irritation to the side affected. Blisters are the most efficacious for this purpose, but iodine or a mild mercurial ointment is often used with advantage. When the pleural surfaces come again into contact, the pain thereby caused is best relieved by strapping the side in the manner suggested by Dr. Roberts. While absorption is in progress it is generally advisable to give a mixture containing iodide of potassium and quinine combined with a bitter. Later, mineral acids with iron and quinine are most useful, and a change of air is to be recommended. In weakly subjects, provided pyrexia be absent, tonic treatment with cod-liver oil and a full and generous diet is often advisable from an early stage of the disease. Many cases of this kind are seen at the Brompton Consumption Hospital. If after fourteen days no absorption has taken place, the question of performing paracentesis must be taken in consideration. Dr. Douglas Powell lays down the following rules with regard to the question of operation: (i.) That operative treatment is not called for if the skodaic resonance be good down to the third rib; (ii.) that the continuance of pyrexia at the end of the second week would be against immediate operative interference. If, however, no absorption had taken place by the end of the third week a portion of the fluid may be removed; (iii.) that early interference is advisable in those cases with a bad family history; (iv.) that if skodaic resonance rises up to or above the second rib paracentesis is called for; (v.) that paracentesis is necessary if the fluid be purulent. Clinically the following conditions call for early interference: (i.) Great dyspnœa or palpitation; (ii.) signs of engorgement of the opposite lung; (iii.) if the fluid has accumulated with great rapidity; (iv.) if disease of the heart exists. If paracentesis is found to be necessary, the operation is best performed with the siphon aspirator. The site of puncture will depend on the position of the fluid, but in the absence of any special indication Dr. Powell prefers the sixth space in the mid axillary line.

*Method of Performing Paracentesis.*—The patient should lie near the edge of the bed with his head and shoulders slightly raised. The side to be operated on should be carefully washed. The aspirating needle should be made surgically clean. This is best done by placing the needle in a test tube, which is filled with water, and then boiled for some minutes, after which the needle is placed in carbolic solution (1·20). The spot chosen for puncture should be made anæsthetic by the application of ice, or the subcutaneous injection of a few drops of a six per cent. solution of cocaine. A preliminary incision through the skin with the knife is recommended by Dr. Powell, and the needle should be smartly thrust into the pleural cavity. It is important that the apparatus should be proved to be in working order before paracentesis is done. The fluid should be withdrawn slowly and completely, unless severe cough supervenes, when it is advisable to terminate the operation. When the instrument is withdrawn care must be taken that no air enters the pleural cavity. The point of puncture is best covered with a pad of lint previously soaked in collodion, which is kept in position by means of a piece of strapping. The subcutaneous injection of 1-5 grain of morphia relieves the coughing that sometimes follows the operation.

